# Supplementary material for: Iterative improvement in the automatic modular design of robot swarms
Source: PeerJ Comput Sci. 2020 Dec 7;6:e322. doi: 10.7717/peerj-cs.322 (PMC7924708; doi:10.7717/peerj-cs.322)
Supplement: Supplemental Information 3 [file peerj-cs-06-322-s003.zip › argos3/doc/api/standalone/a00639_source.html]

ARGoS: plugins/simulator/visualizations/qt-opengl/qtopengl\_joystick.cpp Source File


- Main Page
- Related Pages
- Namespaces
- Classes
- Files

- File List
- File Members

# plugins/simulator/visualizations/qt-opengl/qtopengl\_joystick.cpp

Go to the documentation of this file.

```
00001 
00010 #include "qtopengl_joystick.h"
00011 #include <argos3/core/utility/configuration/argos_exception.h>
00012 #include <argos3/core/utility/logging/argos_log.h>
00013 
00014 namespace argos {
00015 
00016    /****************************************/
00017    /****************************************/
00018 
00019    CQTOpenGLJoystick::CQTOpenGLJoystick(QObject *parent, int joystickEventTimeout, bool doAutoRepeat, int repeatDelay)
00020       : QObject(parent)
00021    {
00022       if ( SDL_Init(SDL_INIT_JOYSTICK) == 0 ) {
00023          int i;
00024          for (i = 0; i < SDL_NumJoysticks(); i++) {
00025             joystickNames.append(SDL_JoystickName(i));
00026             LOG << "[INFO] Found joystick #" << i << ": \"" << SDL_JoystickName(i) << "\"" << std::endl;
00027          }
00028          connect(&joystickTimer, SIGNAL(timeout()), this, SLOT(processEvents()));
00029       } else {
00030          THROW_ARGOSEXCEPTION("QTOpenGLJoystick: couldn't initialize SDL joystick support");
00031       }
00032 
00033       joystick = NULL;
00034       numAxes = numButtons = numHats = numTrackballs = 0;
00035       autoRepeat = doAutoRepeat;
00036       autoRepeatDelay = repeatDelay;
00037       eventTimeout = joystickEventTimeout;
00038    }
00039 
00040 /****************************************/
00041 /****************************************/
00042 
00043    CQTOpenGLJoystick::~CQTOpenGLJoystick()
00044    {
00045       if ( isOpen() )
00046          close();
00047 
00048       SDL_Quit();
00049    }
00050 
00051    /****************************************/
00052    /****************************************/
00053 
00054    void CQTOpenGLJoystick::open(int stick)
00055    {
00056       if ( isOpen() )
00057          close();
00058 
00059       joystick = SDL_JoystickOpen(stick);
00060       if ( joystick ) {
00061          numAxes = SDL_JoystickNumAxes(joystick);
00062          numButtons = SDL_JoystickNumButtons(joystick);
00063          numHats = SDL_JoystickNumHats(joystick);
00064          numTrackballs = SDL_JoystickNumBalls(joystick);
00065          joystickTimer.start(eventTimeout);
00066       } else {
00067          THROW_ARGOSEXCEPTION("QTOpenGLJoystick: couldn't open SDL joystick #%d" << stick);
00068       }
00069    }
00070 
00071    /****************************************/
00072    /****************************************/
00073 
00074    void CQTOpenGLJoystick::close()
00075    {
00076       joystickTimer.stop();
00077       if ( joystick )
00078          SDL_JoystickClose(joystick);
00079       joystick = NULL;
00080       numAxes = numButtons = numHats = numTrackballs = 0;
00081    }
00082 
00083    /****************************************/
00084    /****************************************/
00085 
00086    void CQTOpenGLJoystick::processEvents()
00087    {
00088       if ( !isOpen() )
00089          return;
00090 
00091       SDL_JoystickUpdate();
00092 
00093       int i;
00094       for (i = 0; i < numAxes; i++) {
00095          Sint16 moved = SDL_JoystickGetAxis(joystick, i);
00096          if ( abs(moved) >= deadzones[i] ) {
00097             if ( (moved != axes[i]) ) {
00098                int deltaMoved = abs(axes[i] - moved);
00099                if ( deltaMoved >= sensitivities[i] )
00100                   emit axisValueChanged(i, moved);
00101                axes[i] = moved;
00102                axisRepeatTimers[i].restart();
00103             } else if (autoRepeat && moved != 0) {
00104                if ( axisRepeatTimers[i].elapsed() >= autoRepeatDelay ) {
00105                   emit axisValueChanged(i, moved);
00106                   axes[i] = moved;
00107                }
00108             } else
00109                axisRepeatTimers[i].restart();
00110          } else
00111             emit axisValueChanged(i, 0);
00112       }
00113       for (i = 0; i < numButtons; i++) {
00114          Uint8 changed = SDL_JoystickGetButton(joystick, i);
00115          if ( (changed != buttons[i]) ) {
00116             emit buttonValueChanged(i, (bool) changed);
00117             buttons[i] = changed;
00118             buttonRepeatTimers[i].restart();
00119          } else if (autoRepeat && changed != 0) {
00120             if ( buttonRepeatTimers[i].elapsed() >= autoRepeatDelay ) {
00121                emit buttonValueChanged(i, (bool) changed);
00122                buttons[i] = changed;
00123             }
00124          } else
00125             buttonRepeatTimers[i].restart();
00126       }
00127       for (i = 0; i < numHats; i++) {
00128          Uint8 changed = SDL_JoystickGetHat(joystick, i);
00129          if ( (changed != hats[i]) ) {
00130             emit hatValueChanged(i, changed);
00131             hats[i] = changed;
00132             hatRepeatTimers[i].restart();
00133          } else if (autoRepeat && changed != 0) {
00134             if ( hatRepeatTimers[i].elapsed() >= autoRepeatDelay ) {
00135                emit hatValueChanged(i, changed);
00136                hats[i] = changed;
00137             }
00138          } else
00139             hatRepeatTimers[i].restart();
00140       }
00141 
00142       for (i = 0; i < numTrackballs; i++) {
00143          int dx, dy;
00144          SDL_JoystickGetBall(joystick, i, &dx, &dy);
00145          if ( dx != 0 || dy != 0 )
00146             emit trackballValueChanged(i, dx, dy);
00147       }
00148    }
00149 
00150    /****************************************/
00151    /****************************************/
00152 
00153    int CQTOpenGLJoystick::getAxisValue(int axis)
00154    {
00155       if ( isOpen() ) {
00156          SDL_JoystickUpdate();
00157          return SDL_JoystickGetAxis(joystick, axis);
00158       } else
00159          return 0;
00160    }
00161 
00162    /****************************************/
00163    /****************************************/
00164 
00165 }
```

---

Generated on 10 Jul 2018 for ARGoS by 
 1.6.1
